# e-Graphene: A Computational Platform for the Prediction of Graphene-Based Drug Delivery System by Quantum Genetic Algorithm and Cascade Protocol

**DOI:** 10.3389/fchem.2021.664355

**Published:** 2021-05-07

**Authors:** Suqing Zheng, Jun Xiong, Lei Wang, Dong Zhai, Yong Xu, Fu Lin

**Affiliations:** ^1^School of Pharmaceutical Sciences, Wenzhou Medical University, Wenzhou, China; ^2^Chemical Biology Research Center, Wenzhou Medical University, Wenzhou, China; ^3^Institute of Frontier and Interdisciplinary Science, Shandong University, Qingdao, China; ^4^Center of Chemical Biology, Guangzhou Institute of Biomedicine and Health, Chinese Academy of Sciences, Guangzhou, China

**Keywords:** graphene, graphene-based drug delivery system, GDDS, prediction, quantum genetic algorithm, cascade protocol, QGA

## Abstract

Graphene, as a novel category of carbon nanomaterials, has attracted a great attention in the field of drug delivery. Due to its large dual surface area, graphene can efficiently load drug molecules with high capacity *via* non-covalent interaction without chemical modification of the drugs. Hence, it ignites prevalent interests in developing a new graphene/graphene oxide (GO)-based drug delivery system (GDDS). However, current design of GDDS primarily depends on the prior experimental experience with the trial-and-error method. Thus, it is more appealing to theoretically predict possible GDDS candidates before experiments. Toward this end, we propose to fuse quantum genetic algorithm (QGA) and quantum mechanics (QM)/semi-empirical quantum mechanics (SQM)/force field (FF) to globally search the optimal binding interaction between the graphene/GO and drug in a given GDDS and develop a free computational platform “e-Graphene” to automatically predict/screen potential GDDS candidates. To make this platform more pragmatic for the rapid yet relatively accurate prediction, we further propose a cascade protocol *via* firstly conducting a fast QGA/FF calculation with fine QGA parameters and automatically passing the best chromosomes from QGA/FF to initialize a higher level QGA/SQM or QGA/QM calculation with coarse QGA parameters (e.g., small populations and short evolution generations). By harnessing this platform and protocol, systematic tests on a typical GDDS containing an anticancer drug SN38 illustrate that high fabrication rates of hydroxyl, epoxy, and carboxyl groups on a pristine graphene model will compromise the stability of GDDS, implying that an appropriate functionalization rate is crucial for the delicate balance between the stability and solubility/biocompatibility of GDDS. Moreover, automatic GDDS screen in the DrugBank database is performed and elicits four potential GDDS candidates with enhanced stability than the commonly tested GDDS containing SN38 from the computational point of view. We hope that this work can provide a useful program and protocol for experimental scientists to rationally design/screen promising GDDS candidates prior to experimental tests.

## Introduction

Pristine graphene, discovered by Geim and Novoselov, is a two-dimensional (2D) nanomaterial consisting of a single layer of carbon atoms (Novoselov et al., 2004). It has gained tremendous interests in various research areas (e.g., energy storage and sensors) due to its unique and impressive electrical, thermal, and mechanical properties (Novoselov et al., 2012; Lightcap and Kamat, 2013; Wassei and Kaner, 2013; El-Kady et al., 2016; Georgakilas et al., 2016; Aditya et al., 2017; Yu et al., 2017). Meanwhile, graphene has large dual surface area and high loading capacity, excellent chemical and mechanical stability, and good solubility and biocompatibility after simple fabrication on the surface, consequently graphene and its common derivatives, such as graphene oxides (GO) have exhibited considerable potential in the drug delivery (Chung et al., 2013; Mao et al., 2013; Yang et al., 2013; Goenka et al., 2014; Xianfeng and Feng, 2014; Reina et al., 2017; Ghosal and Sarkar, 2018; Mohammad Omaish et al., 2019).

In 2008, Dai group reported the first case of using GO as a nanocarrier to transport an anticancer drug SN38 (Liu et al., 2008). In their work, they demonstrated that SN38 indeed binds non-covalently with GO to form a stable GO/SN38 complex that affords remarkable activity with IC_50_ of about 6 nM for HCT116 cell line; hence, this pioneering work opens up a novel application of graphene/GO in the field of drug delivery system. Since then, more and more research works on the non-covalent graphene/GO-based drug delivery system (GDDS) are surging and summarized in the comprehensive reviews (Sun et al., 2008; Yang et al., 2008, 2016; Pan et al., 2012; Liu et al., 2013; McCallion et al., 2016; Shim et al., 2016; Zhang et al., 2017; Yi et al., 2019). However, current design of GDDS still highly relies on the traditional trial-and-error approach. Hence, it will be more efficient if GDDS can be evaluated or predicted with the computational methods before experiments.

Regarding the computational predictions of GDDS, molecular dynamics (MD) simulation and quantum mechanics (QM) programs are powerful to accurately investigate the non-bonded interaction between the graphene/GO and ligand for a given GDDS (Gráfová et al., 2010; Ramraj and Hillier, 2010; Calero et al., 2013; Cho et al., 2013; Guo et al., 2013; Vovusha et al., 2013, 2018; Mudedla et al., 2014; Vincent and Hillier, 2014; Wang et al., 2015; Mahdavi et al., 2016, 2020; Krepel and Hod, 2017; Safdari et al., 2017; Ajala et al., 2019; Alkathiri et al., 2019; Azhagiya Singam et al., 2019; Mason et al., 2019). However, both MD and QM methods are usually time-consuming, need to know a reasonable initial binding mode between the graphene/GO and ligand before calculations and tend to entrap the graphene/GO and ligand in a local minimum. To increase the chance of escaping from the local minimum, meta-dynamics (MTD) simulation method (Laio and Parrinello, 2002) is one of the most popular enhanced sampling methods (Bernardi et al., 2015) and extensively employed to promote the crossing of high energetic barriers *via* gradual additions of the Gaussian potential over time. According to the MTD method, the Grimme group developed a very useful conformation search program CREST (Bannwarth et al., 2019), where the root-mean-square deviation (RMSD) of a small molecule is adopted as a reaction coordinate (RC) in the MTD simulation, but this RC cannot capture the relative position between the small molecule and graphene/GO. Alternatively, semi-classical MD simulation method was proposed by the Ceotto group to explore various minima on a potential energy surface, which also is not a computationally cheap method and mainly developed for the prediction of molecular spectroscopy (Conte and Ceotto, 2020; Gandolfi et al., 2020). As a result, these methods may be not very suitable for the rapid prediction/screen of GDDS.

Alternatively, the fast molecular docking programs, such as AutoDock (Morris et al., 2009), which were originally designed to globally search the optimal conformations of a small molecule within the pocket of a biological target, can be borrowed for the quick docking of a small molecule to a graphene/GO model if appropriate parameters for the graphene/GO can be provided. Nevertheless, native scoring functions in these docking programs, initially derived from protein/ligand binding affinities, are also not dedicated to the prediction of drug/graphene (or GO) binding interaction. In short, these molecular docking programs seem not specialized for the accurate prediction/screen of GDDS. Therefore, a single method, such as pure MD, QM, or molecular docking, probably is not the best choice for the fast yet relatively accurate prediction/screen of GDDS, and combining the advantages of some of these methods would be more promising.

**Figure 1:**
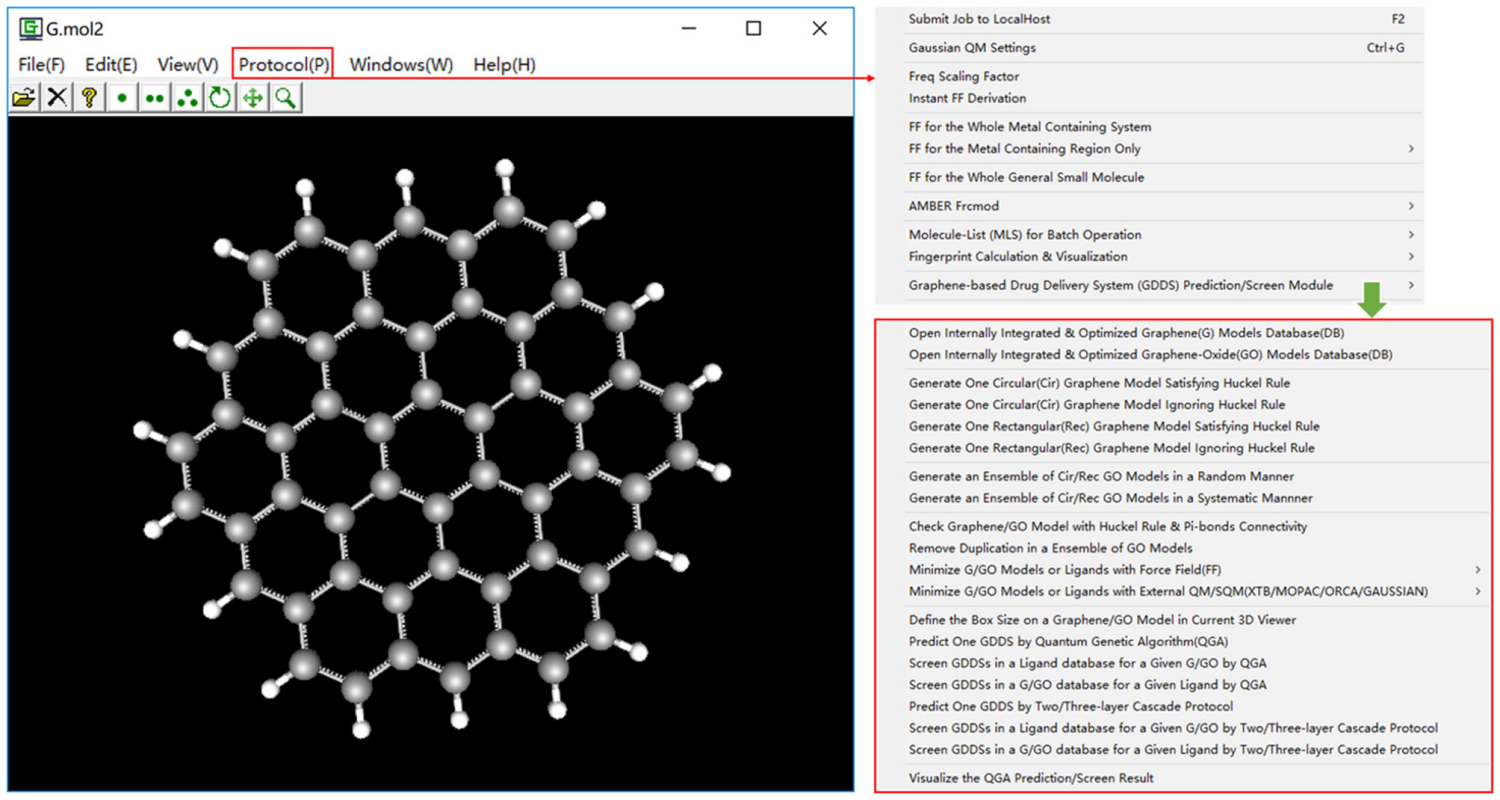
The main interface of e-Graphene program for the automatic prediction/screen of graphene-based drug delivery system (GDDS) by quantum genetic algorithm (QGA) and cascade protocol.

So far there are no dedicated solutions for the prediction/screen of GDDS. Toward this aim, in this work, we propose to fuse quantum genetic algorithm (QGA) (Narayanan and Moore, 1996) and QM/semi-empirical quantum mechanics (SQM)/force field (FF) to predict the optimal drug/graphene (or GO) interaction and further develop a specialized computational platform “e-Graphene” (Figure 1) to predict/screen the potential GDDS in an automatic fashion, which was implemented on the basis of our previous visual FF derivation toolkit “VFFDT” (Zheng et al., 2016). To well balance the speed and accuracy of GDDS prediction/screen, we further suggest an efficient cascade protocol: (1) firstly perform a cheap QGA/FF calculation with fine QGA parameters (e.g., larger populations and longer evolution generations); and (2) automatically pass the best chromosomes from QGA/FF to evoke a higher level QGA/SQM or QGA/QM calculation with coarse QGA parameters (e.g., smaller populations and shorter evolution generations) and also implement this protocol in e-Graphene. To the best of our knowledge, the QGA method and cascade protocol were never used to predict/screen GDDS.

Based on this proposed computational platform and protocol, we organize “Methods” and “Discussion” sections as follows. First, the implementation of main functions in e-Graphene program is briefly introduced in “Methods” section. Second, extensive tests on an exemplary GDDS containing an anticancer drug SN38, which was reported in Liu et al. (2008), are conducted to investigate the impact of different graphene-functionalization rates and sites on the stability of GDDS in “Discussion” section. Finally, drug molecules from DrugBank database (https://go.drugbank.com/) (Wishart et al., 2017) are used to computationally screen potential GDDS candidates for a pristine graphene model, and their results are fully summarized and analyzed in “Discussion” section.

## Methods

In e-Graphene program, six main functions have been implemented and listed as follows: (1) generate graphene/GO models with different sizes and shapes, which can be intuitively visualized in three-dimensional (3D) viewer of e-Graphene; (2) inspect whether graphene/GO models satisfy the Hückel's rule (4n + 2) and full connectivity of Pi-bonds; (3) remove the duplicated GO models; (4) minimize graphene/GO models or ligands by the use of the natively implemented Tripos FF or external QM/SQM programs; (5) develop GDDS prediction/screen functions based on QGA and FF/SQM/QM; (6) implement an automatic and efficient cascade protocol for the GDDS prediction/screen; and (7) visualize GDDS prediction/screen results (Clark et al., 1989). For the sake of conciseness, each function is succinctly introduced one by one.

### Generation and Inspection of Circular/Rectangular Graphene/Graphene Oxide Models

For different computational methods, users have to generate distinct graphene models with different sizes and shapes. e.g., in the QM studies, users often adopt a small circular graphene model satisfying the Hückel's rule (4n + 2). For the convenience of users, four options for the automatic generation of a pristine graphene model have been implemented in e-Graphene.

**Figure 2:**
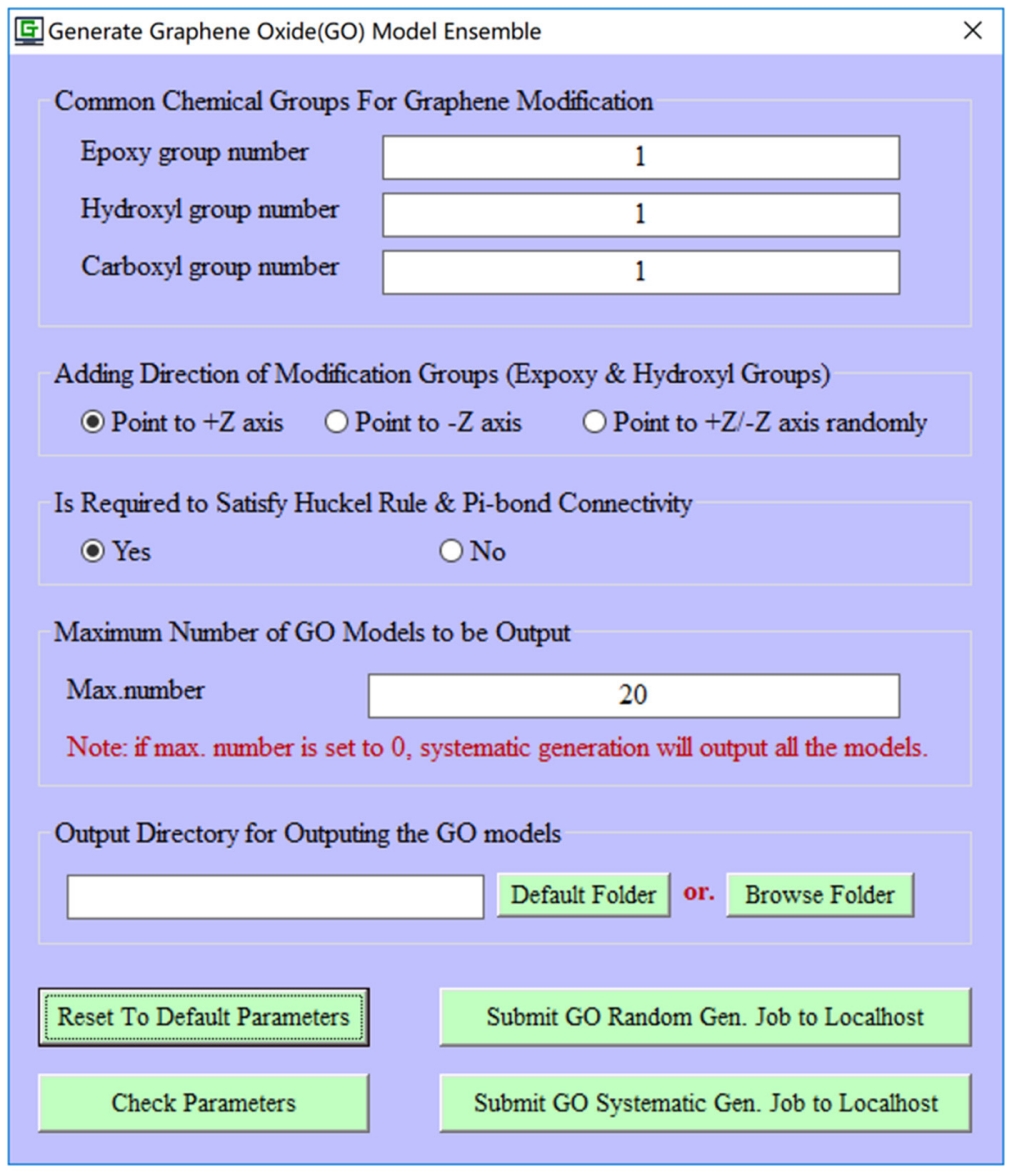
Automatic generation of an ensemble of graphene oxide (GO) models for a given pristine graphene model.

To improve the biocompatibility and solubility, pristine graphene is usually subjected to the chemical modification to become the GO material. However, the precise control of exact functionalization sites/rates on the pristine graphene is still quite challenging in the experiment. Therefore, an ensemble of GO models is generated to approximately stand for the fabricated GO material in the computational study. In our current implementation, common chemical modification groups (hydroxyl, epoxy, and carboxyl groups) can be automatically added to a pristine graphene model. Furthermore, the number of decoration groups (hydroxyl, epoxy, and carboxyl groups), functionalization direction (upper/lower/dual-surface), modification manner (random/systematic manner), and maximum number of GO models (Figure 2) can be specified by users. It is worth mentioning that for each GO model, e-Graphene can automatically inspect both the Hückel's rule (4n + 2) and full connectivity of Pi-bonds for GO models to ensure that the produced GO models still preserve the aromatic property.

### Elimination of Duplicated Graphene Oxide Models

An ensemble of GO models can be automatically enumerated in a random or systematic manner by e-Graphene; however, some produced GO models are actually the same structure after certain translations and rotations. Thus, it is necessary to remove those duplicated GO models from the ensemble before subsequent calculations. In this work, we firstly calculate a large-diameter circular fingerprint “2048bit-ECFP10,” which is one type of commonly used extended connectivity fingerprint (ECFP) (Rogers and Hahn, 2010) to represent each GO model and then eliminate the duplication if some GO models share the same ECFP fingerprint, which has been implemented on the basis of our previous work about machine learning-guided bitterant prediction (Zheng et al., 2018).

### Minimization of Graphene/Graphene Oxide Models

Before the GDDS prediction/screen, all the generated graphene/GO models are usually subjected to the minimization. For the rapid optimization, we have implemented Tripos FF in e-Graphene to automatically minimize the models by Powell minimization method that is natively coded in e-Graphene. At the same time, we also have developed an interface to call the external QM/SQM programs, such as Gaussian03/09/16 (Frisch et al., 2016), ORCA4.2 (Neese, 2012), MOPAC2009/2012/2016 (Stewart, 2016), and XTB6.3 (Bannwarth et al., 2019) to further minimize the graphene/GO model in a higher level of accuracy. In order to minimize all the graphene/GO models in batch mode, automatic calculation of the total formal charge for each model is developed in e-Graphene, whereas the multiplicity for each model is set to 1 by default due to our current focus on the ground state of the graphene/GO model. It is of note that this module in e-Graphene can also be utilized to minimize general small molecules.

### Implementation of QGA-Guided Prediction/Screen of GDDS

**Figure 3:**
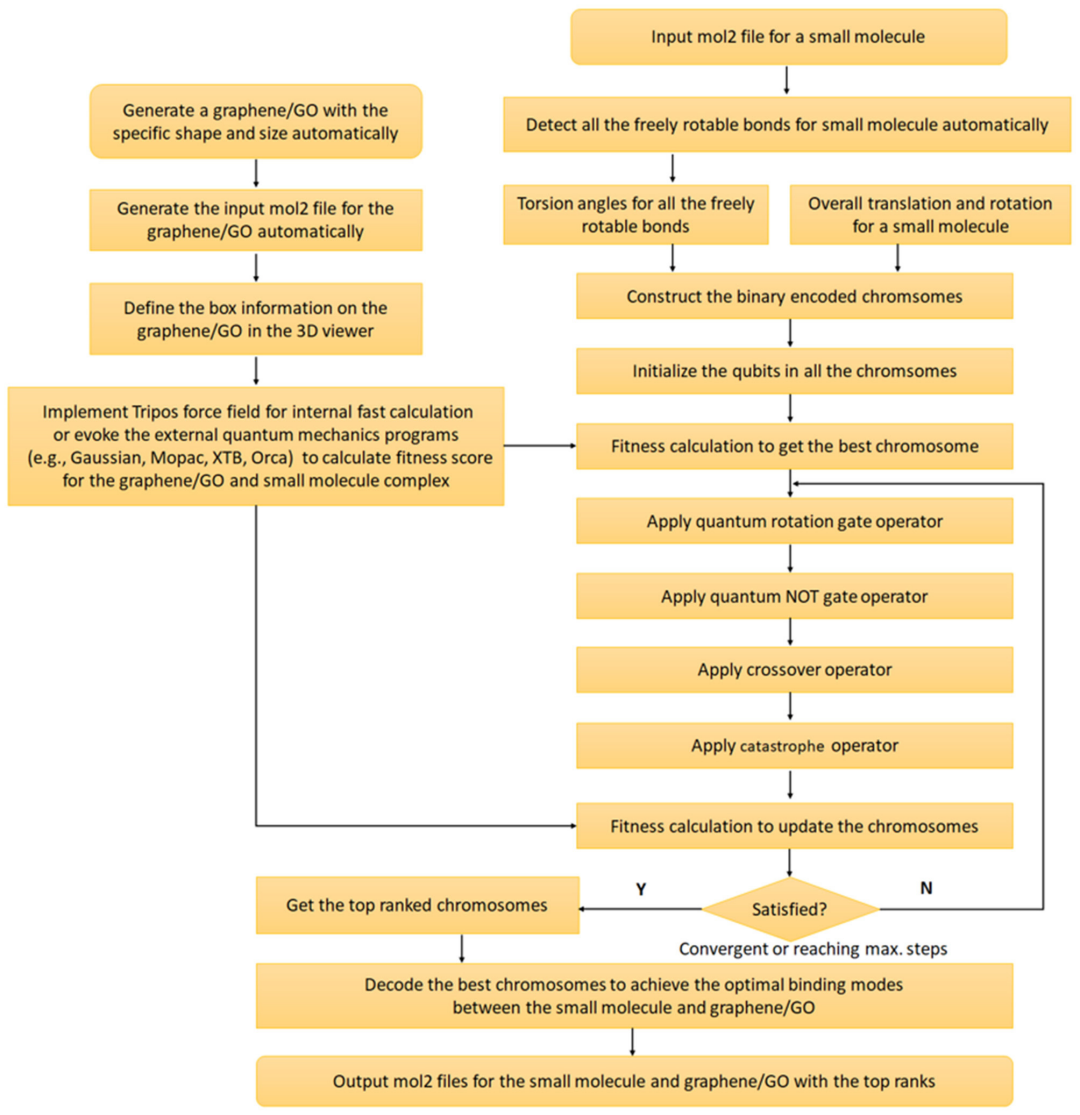
Implementation workflow of the quantum genetic algorithm (QGA) guided prediction of graphene-based drug delivery system (GDDS).

Quantum genetic algorithm was proposed by Narayanan and Moore for the first time in 1996 (Narayanan and Moore, 1996) and introduced the concepts from quantum computation to the genetic algorithm. However, QGA was never used to predict the GDDS before. Thus, we combine QGA and QM/SQM/FF for this purpose. The whole implementation workflow of QGA is displayed in Figure 3, and the key steps are detailed as follows.

First, all the freely rotatable bonds (except the single bonds in the terminal groups, such as methyl group) for a small molecule are automatically detected and recorded, and all the torsion angles of the freely rotatable bonds, besides the overall translation (three variables) and rotation (quaternions with four variables) of the small molecule, are firstly encoded in a chromosome with the form of real number. Then this real-coded chromosome, reflecting one specific conformation of the small molecule, is further converted to a binary-coded chromosome for the subsequent QGA.

**Figure 4:**
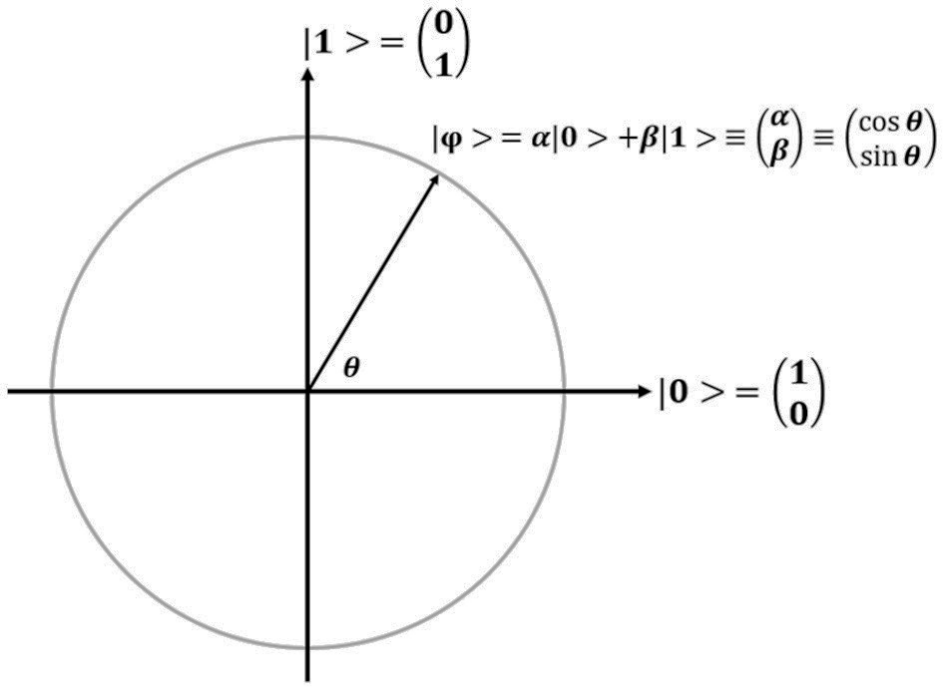
Schematic illustration of a qubit. For the convenience of description, a qubit |ϕ > is simply denoted by (α, β) or (*cos*θ, *sin*θ).

In QGA, each bit in each chromosome is referred to as a qubit, which is the basic unit of information in a quantum computer, and the corresponding state of each qubit is formulated by Dirac notation |ϕ > = α|0> +β|1> with the constraint of |α|^2^+|β|^2^=1, where α and β refer to the probability amplitudes of a qubit (Figure 4). For the simple description, each qubit is denoted by (α, β) or (cosθ, sinθ). Obviously, each qubit can be in the ground state (|0> or |1>), or the quantum superposition state of both ground states. Once a qubit is measured, it will be collapsed to the ground state |0> (or |1>) with a probability of |α|^2^ (or |β|^2^). If |α|^2^ (or |cosθ|^2^) is larger than |β|^2^ (or |sinθ|^2^), the qubit will be degraded to the ground state |0>, otherwise, it will be collapsed to the ground state |1>. Therefore, the concept “qubit” introduced in QGA provides the vast searching space.

Before the evolution of each chromosome, each qubit (*cos*θ, *sin*θ) in each chromosome is initialized to [*cos(*π*/4), sin(*π*/4)*], indicating that all the qubits initially have the same probability of being collapsed to either ground state |0> or |1>. If a randomly generated floating number between 0.0 and 1.0 is less than | cos(π*/4*) |^2^, the qubit will be degraded to the bit “1” (or |1>), otherwise, it will be collapsed to the bit “0” (or |0>). After the measurement of all the qubits in a chromosome, each chromosome is transformed to a conventional binary string containing only the bit “1/0,” which can be further decoded for the fitness calculation to update each chromosome for the next round of evolution.

Quantum rotation gate operator (QRGO) is utilized to update each chromosome with the rotation angle (Δθ) by comparing with the best chromosome in the whole population (Li et al., 2018), which has been defined by Equation (1). All the qubits in each chromosome are updated according to Equation (2), and the detailed update scheme of quantum rotation angle (Δθ) is tabulated in Supplementary Table 1 (Li et al., 2018). Similarly, quantum NOT gate operator (QNGO) is exerted to transform the qubits (Equations 3, 4) for the mutation of a chromosome. In addition, crossover operator is also applied to two different chromosomes to exchange parts of qubits in one chromosome with the corresponding qubits in the other chromosome. Moreover, catastrophe operator is used to reinitialize all the qubits in all the chromosomes if the whole population is trapped in a local minimum without any change of average fitness score in certain successive steps. After applying different operators with certain probabilities, all the newly updated chromosomes are decoded for the fitness calculation and the best chromosome are updated accordingly.

(1) $$\begin{array}{l} QRGO\left(\Delta \theta \right)=\left(\begin{array}{cc} cos\Delta \theta & \;-sin\Delta \theta \\ sin\Delta \theta & \;cos\Delta \theta \end{array}\right) \end{array}$$

where *QRGO* refers to quantum rotation gate operator and Δθ is the variation of quantum rotation angle.

(2) $$\begin{array}{l} \left(\begin{array}{c} \alpha^{\prime }\\ \beta^{\prime } \end{array}\right)=QRGO\left(\Delta \theta \right)\left(\begin{array}{c} \alpha \\ \beta \end{array}\right)=\left(\begin{array}{cc} cos\Delta \theta & \;-sin\Delta \theta \\ sin\Delta \theta & \;cos\Delta \theta \end{array}\right)=\left(\begin{array}{c} \alpha \\ \beta \end{array}\right)\\ \;=\left(\begin{array}{c} cos\left(\theta +\Delta \theta \right)\\ sin\left(\theta +\Delta \theta \right) \end{array}\right) \end{array}$$

where each qubit is updated according to the *QRGO*; α and β are the probability amplitudes of the original qubit; and α′ and β′ are the probability amplitudes of the updated qubit.

(3) $$\begin{array}{l} QNGO=\left(\begin{array}{cc} 0 & \;1\\ 1 & \;0 \end{array}\right) \end{array}$$

where *QNGO* refers to quantum NOT gate operator.

(4) $$\begin{array}{l} \left(\begin{array}{c} \alpha^{\prime }\\ \beta^{\prime } \end{array}\right)=\left(\begin{array}{cc} 0 & \;1\\ 1 & \;0 \end{array}\right)\left(\begin{array}{c} \alpha \\ \beta \end{array}\right)=\left(\begin{array}{c} \beta \\ \alpha \end{array}\right) \end{array}$$

where each qubit is updated according to the *QNGO*; α and β refer to the probability amplitudes of the original qubit; and α′ and β′ are the probability amplitude of the updated qubit.

Fitness of each chromosome is calculated from the potential energy of GDDS (Equation 5) or binding energy between the graphene (or GO) and ligand by Equations (6, 7). The potential energy or binding energy is computed by using the natively implemented Tripos FF in e-Graphene for the fast prediction or evoking the external QM/SQM programs for more accurate prediction.

(5) $$\begin{array}{l} \text{Fitness}=-1.0\times E_{GDDS} \end{array}$$

(6) $$\begin{array}{l} \text{Fitness}=-1.0\times E_{Delta} \end{array}$$

(7) $$\begin{array}{l} E_{Delta}=E_{GDDS}-E_{G}-E_{lig} \end{array}$$

where *E*_*GDDS*_, *E*_*G*_, and *E*_*lig*_ are the potential energies of a given GDDS conformation, graphene/GO, and ligand, respectively. *E*_*Delta*_ refers to the binding energy between the graphene/GO and ligand in a given GDDS conformation. The potential energy can be calculated by the internally implemented Tripos FF or externally evoked SQM/QM programs, such as Gaussian03/09/16, ORCA4.2, MOPAC2009/2012/2016, and XTB6.3. It is worth noting that Equation (5) is used to calculate the fitness by default, although both options (Equations 5, 6) are supported in e-Graphene.

QGA run can be terminated if the maximum number of evolution generation is reached or the average fitness difference between certain successive steps are consistently lower than 0.001, which is the default convergence criterion and can be customized by users. Once completed, all the best chromosomes are decoded to return the final conformations with customized Tripos Mol2 format files containing the energy information, which can be visualized by another module in e-Graphene as shown below.

### Implementation of an Efficient Cascade Protocol for the GDDS Prediction/Screen

**Figure 5:**
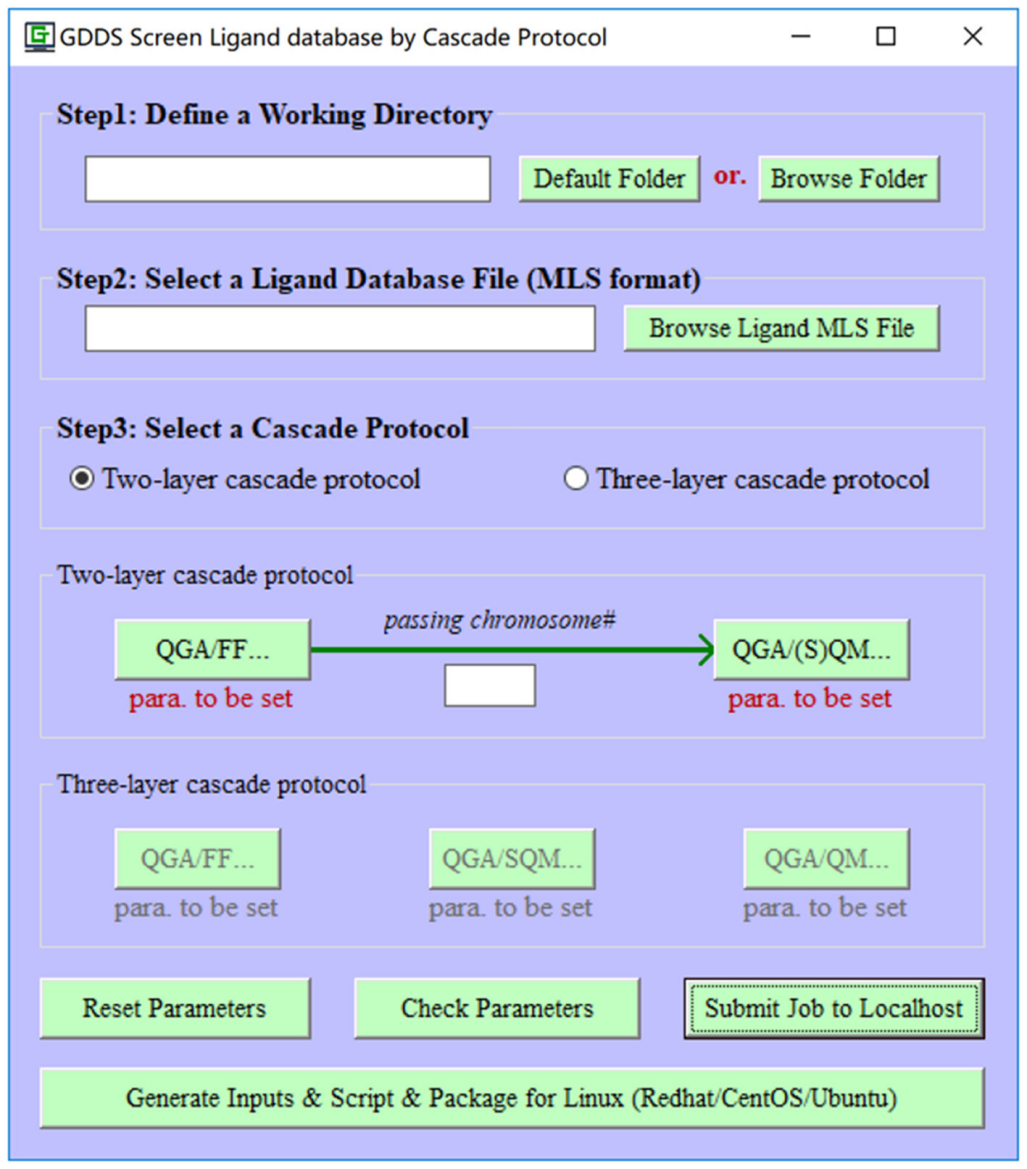
A two-/three-layer cascade protocol implemented in e-Graphene.

To make e-Graphene more efficient, we further develop a cascade protocol for the GDDS prediction/screen. Even though the default two-layer cascade protocol (QGA/FF-QGA/SQM or QGA/FF-QGA/QM) is strongly recommended and extensively evaluated in this work, more computationally expensive three-layer (QGA/FF–QGA/SQM-QGA/QM) cascade protocol is also supported in e-Graphene program (Figure 5). Users can select either protocol, define the relevant parameters for each layer, and set the number of the best chromosomes that are automatically migrating from the previous layer to the next layer for the population initialization of QGA in the next layer. It should be noted that the coarse QGA parameters, such as smaller populations and shorter evolution generations, should be given for the layer with QGA/SQM or QGA/QM in the practical prediction/screen; moreover, solvation and dispersion effects should be considered in the layer with QGA/SQM or QGA/QM.

### Visualization of the GDDS Prediction/Screen Result

**Figure 6:**
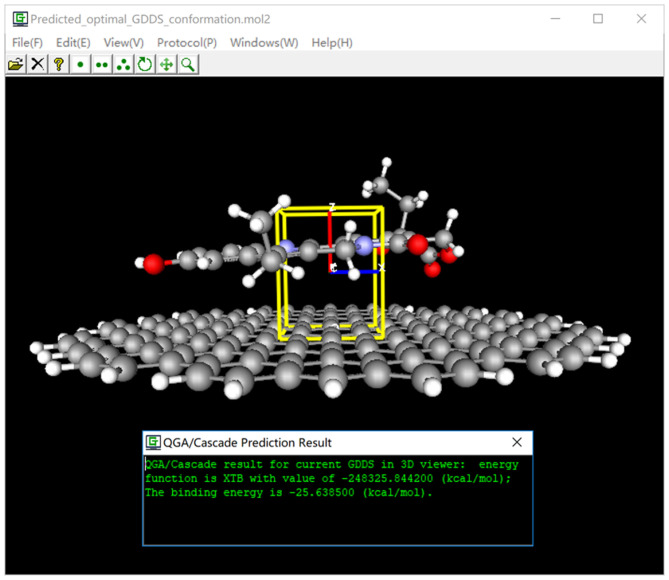
Visualization of graphene-based drug delivery system (GDDS) prediction/screen result.

After GDDS prediction/screen, we are usually very interested in the optimal binding mode/energy between the graphene/GO and ligand for a given GDDS. For this purpose, we have implemented a convenient function to intuitively and synchronously visualize the graphene (or GO)-ligand binding mode/energy, and box information in 3D viewer of e-Graphene (Figure 6) so that users can easily examine whether QGA-guided prediction/screen gives reasonable results before further experimental tests.

## Results and Discussion

In this work, we propose to combine QGA and QM/SQM/FF for the intelligent prediction/screen of GDDS and further put forward an efficient cascade protocol by seamlessly integrating consecutive runs of QGA/FF–QGA/SQM (or QGA/FF–QGA/SQM-QGA/QM). For the convenience of users, we have developed a relatively comprehensive program “e-Graphene” with a graphic user interface to prepare and perform automatic prediction/screen of GDDS with/without the cascade protocol. The whole package including program, manual, and tutorial files is freely available on the Dropbox and Baidu Cloud public shared-folders with the respective download links (https://www.dropbox.com/sh/xj8wnc08kmw8b7a/AAAyXFbObUsk28QwFVfLjXCka?dl=0) and (https://pan.baidu.com/s/15_N7FNPrfjjoaA8f2s5gwQ with the four-letter extraction code: *urdf*). It is worth mentioning that e-Graphene has been fully tested and directly run on the local machine with 64-bit version of Windows10 operating system (OS) and can also prepare GDDS prediction/screen jobs including the input files, scripts, and a binary package for a remote computer with 64-bit version of Redhat5/6/7, CentOS5/6/7, or Ubuntu16.04/18.04/20.04-LTS OS. To demonstrate the applicability of this program and protocol, two typical examples are provided as below.

### Different Chemical Functionalization Rates and Sites Impacting on GDDS Stability

In the work of Liu et al., GO material was demonstrated to effectively carry an anticancer drug SN38 by non-covalent interactions (Liu et al., 2008). However, the GO material is generally assumed to be a mixture from the structural point of view due to the fact that the oxidation process of pristine graphene is quite challenging to be precisely manipulated in terms of the exact functionalization sites and rates; hence, distinct functionalization sites and rates may have certain influence on the stability of GDDS, which was never probed in the original work of Liu et al. (2008). For the clarity, the stability mentioned in this work specifically refers to the binding energy between the graphene/GO and ligand for a given GDDS.

To tentatively explore this problem from the computational perspective, appropriate models should be produced to stand for the pristine and fabricated graphene materials. More concretely, a circular graphene model with the diameter of 21.760 Å and the total atom number of 168 is generated *via* clicking on the menu (Figure 1), and then this model is randomly functionalized by N hydroxyl group(s), N epoxy group(s), and N carboxyl group(s) (*N* = 1, 2, 3, 4, and 5) to produce five sets of 20 GO models, which represent five different rates of chemical modification on the pristine graphene model and are simply denoted by GO-1, GO-2, GO-3, GO-4, and GO-5, respectively, for the convenience of following discussion. Subsequently, one pristine graphene model and 100 GO models are combined to form a graphene/GO model database, which is systematically minimized in batch by evoking an external and efficient SQM program XTB6.3 with the keyword “–*opt –gfn 2 –gbsa water reference*” including the solvation and dispersion effect. Similarly, the 3D structure of SN38 with the neutral form is retrieved from the PubChem database (https://pubchem.ncbi.nlm.nih.gov) and also optimized by XTB6.3 with the same keyword.

In order to investigate the effect of different graphene-functionalization rates and sites on the stability of GDDS including SN38, the two-layer cascade protocol (QGA/FF–QGA/SQM) will be applied to all 101 graphene/GO-SN38 pairs due to our limited computational resource. In this protocol, the full parameter settings for current tests are shown in Supplementary Figures 1–3, while the key parameters will be highlighted as follow: the evolution generation number and population size in the fast QGA/FF calculation are set to 5,000 and 100, respectively, whereas the counterparts in the slow QGA/SQM calculation are set to 500 and 20, respectively. Ten best chromosomes from the cheap QGA/FF calculation are automatically passed to QGA/SQM. SQM is set to XTB6.3 with the keyword “–*gfn 2 –gbsa water reference*” including the solvation and dispersion effect as well. The respective probabilities of QRGO, QNGO, and crossover operation are set to 0.5, 0.1, and 0.7, respectively. Catastrophe operator is applied if the average fitness difference between two consecutive generations is always lower than 0.001 within 50 successive steps during the QGA evolution. The fitness is calculated by Equation (5) for the current test. According to these parameters, this cascade protocol was repeated three times for each GDDS because of the intrinsic randomness in QGA. Therefore, 303 runs with this cascade protocol were conducted, and all the corresponding results for the best chromosomes are listed in Supplementary Table 2.

**Figure 7:**
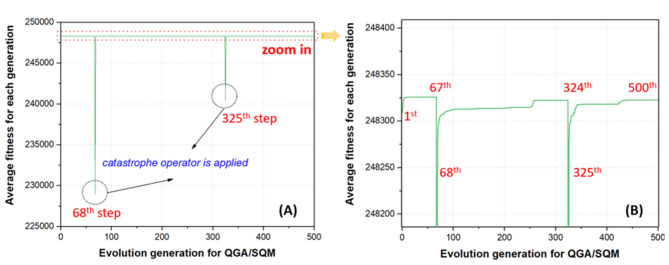
Average fitness for the whole population along the quantum genetic algorithm (QGA)/semi-empirical quantum mechanics (SQM) evolution: **(A)** original curve and **(B)** local enlarged curve.

Before evaluating the impact of different functionalization rates and sites on the stability of GDDS, the convergence analysis was first conducted to inspect whether QGA/FF in our cascade protocol could assist the convergence of QGA/SQM. For the convenience of discussion, one QGA run for the GDDS containing the pristine graphene model and SN38 was taken as an example. The average fitness of the whole chromosomes along the evolution of QGA/SQM is monitored because our final optimal solutions are actually derived from QGA/SQM rather than QGA/FF. In Figure 7, it is clearly shown that catastrophe operator is automatically applied in the 68th and 325th steps, respectively, where the average fitness declines dramatically due to the fact that all the chromosomes are completely reinitialized in order to escape from the previous local minimum. However, the average fitness in the 1st step is much larger than that in the 68th or 325th step because the best chromosomes from QGA/FF are used to seed the initial population for QGA/SQM. Apparently, it is much easier to reach the plateau when QGA/SQM run starts from the 1st step, whereas it takes longer evolution to achieve the convergence if QGA/SQM run is completely randomly reinitialized from the 68th or 325th step. From this perspective, QGA/FF indeed can accelerate the convergence of QGA/SQM, and in the practical predictions, users can turn off the option of removing the catastrophe operator and turn on the option of activating the automatic inspection of convergence in QGA/SQM for the faster calculation, while still preserving the catastrophe operator in QGA/FF to produce diverse optimal chromosomes for the subsequent QGA/SQM in the cascade protocol.

To scrutinize the influence of different functionalization rates on the GDDS stability, the binding energy between the graphene/GO model and SN38 is derived from the best chromosome after running the two-layer (QGA/FF–QGA/SQM) cascade protocol. All the results are listed in Supplementary Table 2 and Table 1. From Table 1, it is shown that the 95% confidence intervals of binding energy for graphene/SN38, GO-1/SN38, GO-2/SN38, GO-3/SN38, GO-4/SN38, and GO-5/SN38 are −25.8118 ± 1.2778, −25.7589 ± 0.5120, −24.1041 ± 1.8056, −23.3682 ± 0.8053, −21.9780 ± 1.2295, and −20.3435 ± 1.0982 (kcal/mol), respectively, which suggest that GO-4/SN38 and GO-5/SN38 are obviously less stable than GO-1/SN38 and GO-2/SN38. Therefore, high functionalization rates compromise the stability of GDDS, indicating that an appropriate rate of fabrication on the pristine graphene is very important to achieve a stable GDDS with good solubility and biocompatibility.

**Table 1:** The 95% confidence intervals of binding energies between the graphene/GO model and SN38 derived from three runs with the two-layer cascade protocol.

**Model name**	**Epoxy group number**	**Hydroxyl group number**	**Carboxyl group number**	**Model number**	**Repeats number**	**95% CI of binding energy with SN38 (kcal/mol)**
Graphene	0	0	0	1	3	−25.8118 ± 1.2778
GO-1	1	1	1	20	3	−25.7589 ± 0.5120
GO-2	2	2	2	20	3	−24.1041 ± 1.8056
GO-3	3	3	3	20	3	−23.3682 ± 0.8053
GO-4	4	4	4	20	3	−21.9780 ± 1.2295
GO-5	5	5	5	20	3	−20.3435 ± 1.0982

*(1) CI is short for confidence intervals; (2) “repeats” means that each cascade protocol is repeated for three times in this work; and (3) the binding energy reported here includes the solvation free energy*.

**Figure 8:**
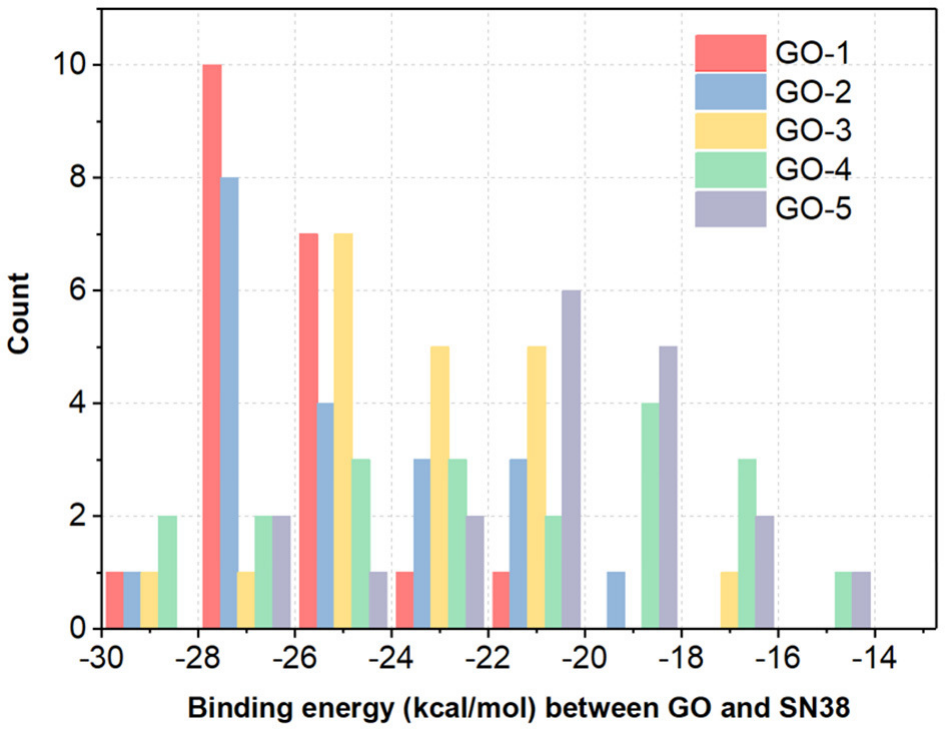
The histogram of average binding energies (kcal/mol) between GO models and SN38. For each GO-X (X = 1, 2, 3, 4, and 5), all the GO-X models have the same functionalization rate with different functionalization sites.

To further evaluate the impact of different functionalization sites on the stability of GDDS with the same fabrication rate, the histogram of binding energies between the GO models and SN38 is as plotted in Figure 8. It is manifested that the binding energies of GOs/SN38 with the same functionalization rate, but different fabrication sites, have large variations. Here, we take the GO-1 models as examples, the lowest and highest binding energy between GO-1 and SN38 are −27.7450 and −22.3940 kcal/mol, respectively. Thus, different functionalization sites have a remarkable impact on the GDDS stability, but unfortunately the exact fine-tuning of functionalization sites on the pristine graphene still remains challenging from the experimental perspective, which indicates that it is more reasonable to harness the GO models with different functionalization sites to stand for the GO material with a certain average functionalization rate.

### GDDS Screen for a Given Graphene/GO Model and a Given Chemical Database

Our cascade protocol can also be used to computationally screen the potential GDDS candidates for a given graphene (or GO) model and a given chemical database. For the demonstration purpose, we adopt the same circular pristine graphene model (diameter: 21.760 Å) as mentioned above and choose the DrugBank database (Wishart et al., 2017) for this screen. More specifically, 3D structures of 8,820 drug molecules with the neutral form are retrieved from the web server of DrugBank and then are subjected to the filtering of ChemAxon LogP larger than 5.0, which affords 200 highly hydrophobic drug molecules. Furthermore, if the longest distance within the 3D structure of a drug molecule is larger than the diameter of our current circular graphene model, this drug molecule will be excluded in our screen because our current graphene model in this demonstration is not large enough and its edge effect will be severe for this molecules. According to this criterion, 121 drug molecules are obtained and further minimized by XTB6.3 program with the keyword “–*opt –gfn 2 –gbsa water reference*,” which are finally utilized for the current screen by the two-layer cascade protocol (QGA/FF–QGA/SQM). SQM is also set to XTB6.3 with the keyword “–*gfn 2 –gbsa water reference*” that takes into account the solvation and dispersion effect. All the parameters for this protocol used in the screen are exactly the same as those in the previous section. This screen campaign was also performed for three times due to the inherent stochastic nature of QGA.

**Figure 9:**
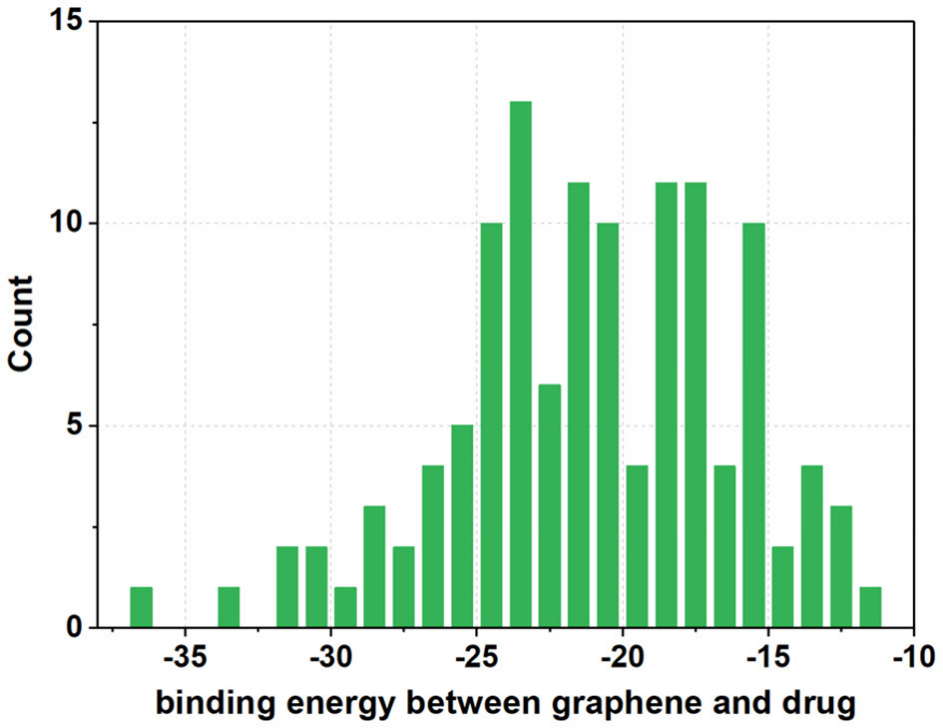
The histogram of average binding energies (kcal/mol) between the graphene model and 121 drugs after screen with the two-layer (QGA/FF–QGA/SQM) cascade protocol.

Once three runs of GDDS screen in e-Graphene were completed, all the conformations for the best chromosomes were harvested and prioritized according to the average binding energy over three repeats (Supplementary Table 3 and Figure 9). Only 12 GDDS candidates (or 12 graphene/drugs) had lower binding energies (−36.3613 ~ −27.6150 kcal/mol) than the 95% CI of binding energy for the graphene model/SN38 (−27.0896, −24.5340) kcal/mol as shown in our previous test. Further manual scrutinization of 12 compounds in the DrugBank database suggested that four of them were anticancer drugs (midostaurin, nilotinib, tucatinib, and arzoxifene) that usually need to be delivered to the specific cancer cells, and their average binding energies with the graphene model were −31.8856, −30.8095, −29.6934, and −27.8303 kcal/mol, respectively (Table 2), whereas most of the remaining drugs for the external usage were not necessary for the delivery by the graphene/GO. Thus, only these four anticancer drugs are proposed to be used for the potential GDDS candidates from the computational perspective.

**Table 2:** Four predicted anticancer drugs for the potential GDDS candidates.

**DrugBank ID**	**Drug Name**	Average binding energy^*^ (kcal/mol)	**Chemical structure**
DB06595	Midostaurin	−31.8856	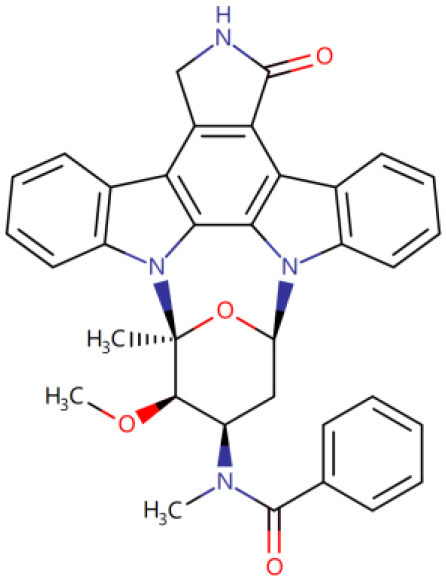
DB04868	Nilotinib	−30.8095	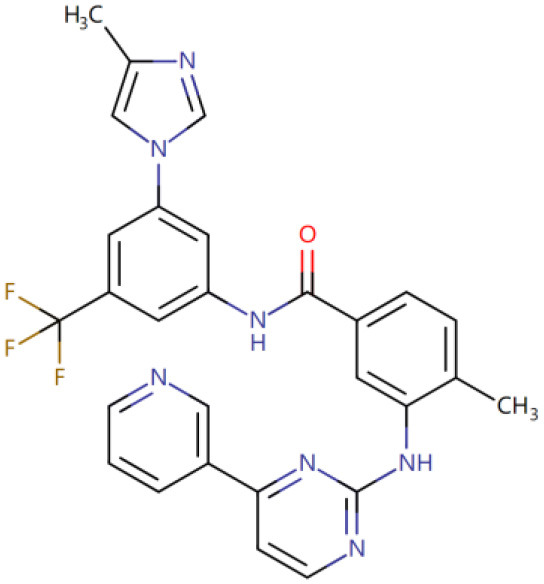
DB11652	Tucatinib	−29.6934	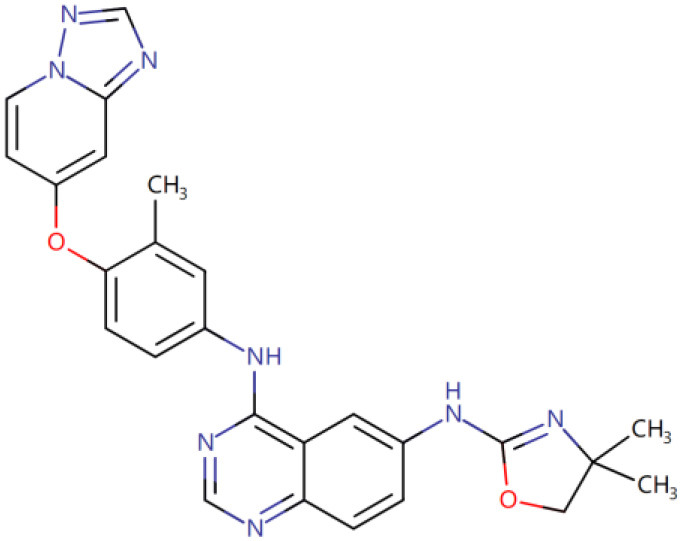
DB06249	Arzoxifene	−27.8303	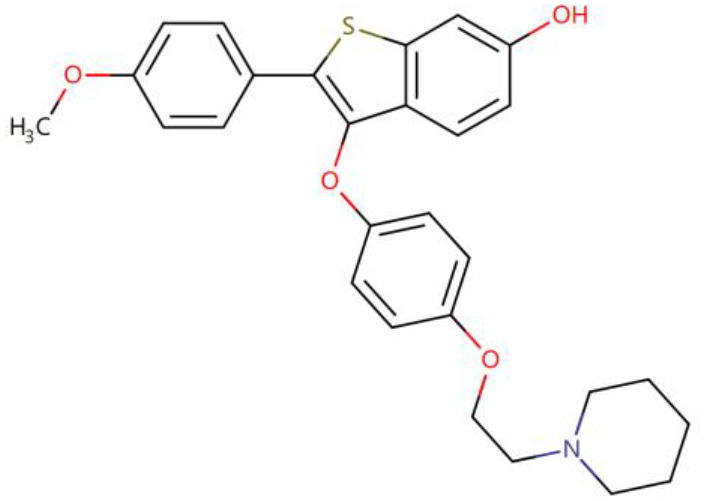

* *The average binding energy is obtained by averaging over three repeats and includes both solvation and dispersion effects*.

### Limitation and Outlook of Our Computational Platform and Protocol

Although e-Graphene could provide a pragmatic computational platform and protocol for the automatic prediction/screen of GDDS based on QGA and QM/SQM/FF, it indeed had several drawbacks: (1) QGA belongs to the stochastic method, which produces different results for different runs. Thus, multiple runs are strongly recommended to achieve more convergent results, which undoubtedly brings some extra computational burden. (2) The speed and accuracy of two-layer (QGA/FF–QGA/SQM) or three-layer (QGA/FF–QGA/SQM–QGA/QM) cascade protocol was largely dependent on the computationally expensive SQM or QM with different keywords and parameters. Careful benchmarks of different choices are highly advised to obtain the comparatively accurate yet fast combination for the practical prediction/screen of GDDS. Thus, it is suggested for users to clearly understand the weakness of e-Graphene before applying it to their own projects.

As recent booming of quantum computation in various areas, QGA possesses intrinsic quantum operators, thus we tentatively envision that if QGA can be ported to the quantum computer simulator and subsequent real quantum computer, QGA might release its tremendous native capacity. However, it still has a very long way to go before QGA-based protocol can be fully deployed in the quantum computer due to the following two main obstacles: (1) the current quantum computers support a small number of qubits, e.g., 53 bits from Google quantum computer (Arute et al., 2019); (2) the accessibility of quantum computers is quite limited, which undoubtedly imposes a steep learning curve, even though quantum computer simulators could alleviate this circumstance to some extent. Despite of those difficulties, we believe that this emerging area still draws more and more researchers to foster its sustainable development.

## Conclusion

In this work, we present a practical computational platform “e-Graphene” for the automatic prediction/screen of GDDS by the seamless integration between QGA and QM/SQM/FF and further propose a cascade protocol for the efficient prediction/screen of GDDS. In this platform, a graphene/GO model can be automatically generated according to the shape and size specified by users, subsequently minimized by the internally implemented Tripos FF or externally evoked QM/SQM programs, and finally used for the preparation of GDDS prediction/screen job with/without the cascade protocol, which can be ran on the local machine with Windows10 and on the remote machine with Red Hat/CentOS/Ubuntu. By using this platform, two representative test cases are adopted to demonstrate its pragmatic functions. One is systematic tests on a extensively used GDDS containing an anticancer drug SN38, which manifests that high rates of functionalization on the pristine graphene reduces the stability of GDDS and also indicates that an appropriate fabrication rate is crucial to attain an optimal GDDS with good stability, solubility, and biocompatibility. The other is the GDDS screen in the DrugBank database for a pristine graphene model, which harvests four potential GDDS candidates with better stability than the common GDDS containing SN38. At last, the limitations of this computational platform are also addressed, although it is envisioned that this free platform could enable experimental scientists to automatically predict possible GDDS candidates before experimental tests.

## Data Availability Statement

The original contributions presented in the study are included in the article/Supplementary Material, further inquiries can be directed to the corresponding authors.

## Author Contributions

SZ and JX performed the calculations and analysis. LW and DZ tested the program. YX and FL revised the manuscript. SZ and FL conceived the workflow, wrote the manuscript, and developed the program. All authors contributed to the article and approved the submitted version.

## Conflict of Interest

The authors declare that the research was conducted in the absence of any commercial or financial relationships that could be construed as a potential conflict of interest.
