# Supplementary material for: e-Graphene: A Computational Platform for the Prediction of Graphene-Based Drug Delivery System by Quantum Genetic Algorithm and Cascade Protocol
Source: Front Chem. 2021 May 7;9:664355. doi: 10.3389/fchem.2021.664355 (PMC8138207; doi:10.3389/fchem.2021.664355)
Supplement: Supplementary file 1 [file Data_Sheet_1.pdf]

Supporting Information

e-Graphene: A Computational Platform for the Prediction of Graphene-based Drug Delivery System by Quantum Genetic Algorithm and Cascade Protocol

Suqing Zheng,[a,b] Jun Xiong,[a] Lei Wang,[a] Dong Zhai,[c] Yong Xu,[d] Fu Lin [a*]*

[a]: School of Pharmaceutical Sciences, Wenzhou Medical University, Wenzhou, Zhejiang, P. R. China, 325035

[b]: Chemical Biology Research Center, Wenzhou Medical University, Wenzhou, Zhejiang, P. R. China, 325035

[c]: Institute of Frontier and Interdisciplinary Science, Shandong University, Qingdao, Shandong, P. R. China, 266237

[d]: Center of Chemical Biology, Guangzhou Institute of Biomedicine and Health, Chinese Academy of Sciences, Guangzhou, Guangdong, P. R. China, 510530

[*]: To whom all correspondence should be addressed. E-mail: lin1449@126.com; zsq_2016@126.com.

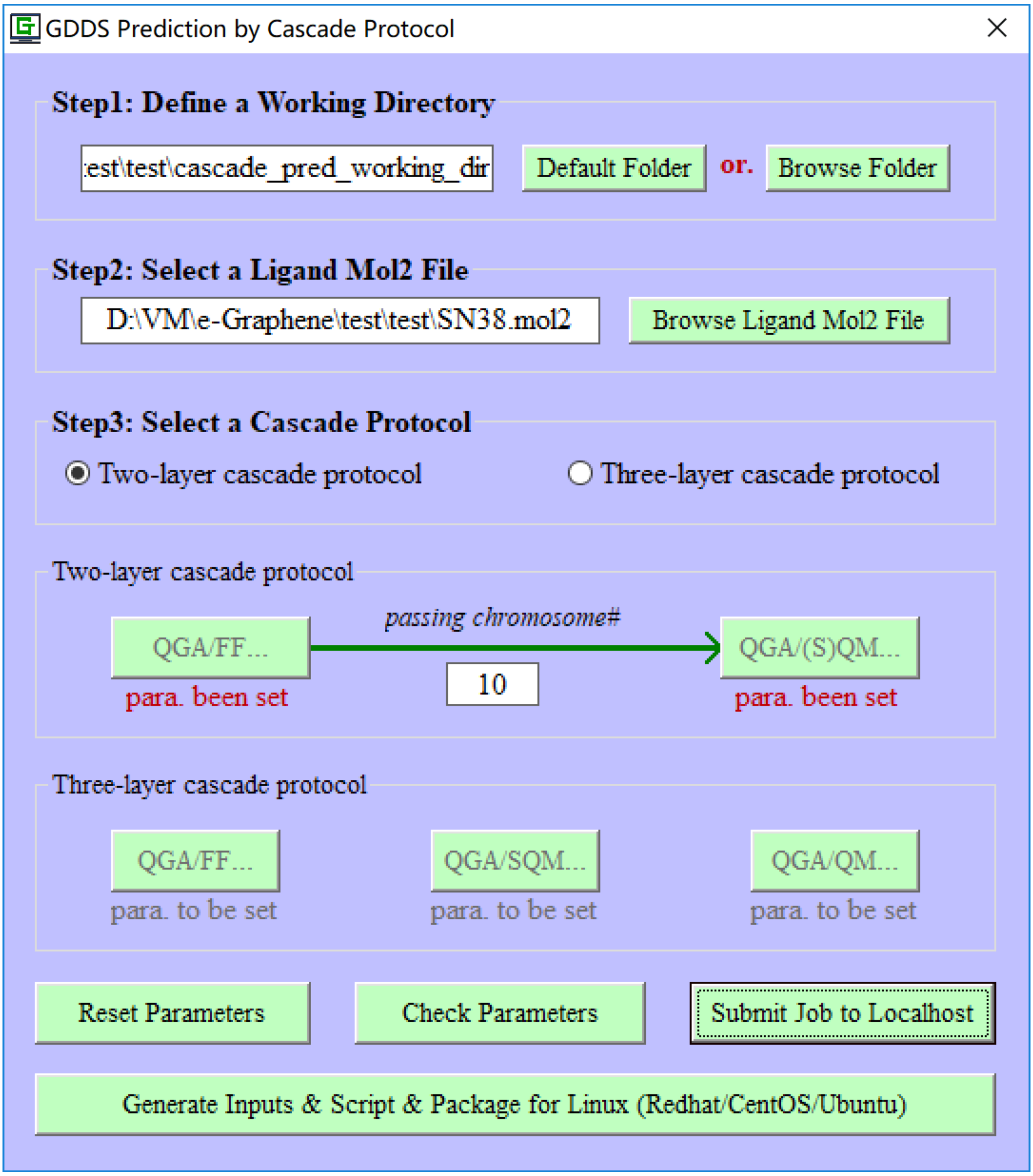

Figure S1. Parameter settings for the two-layer cascade protocol (QGA/FF-QGA/SQM).

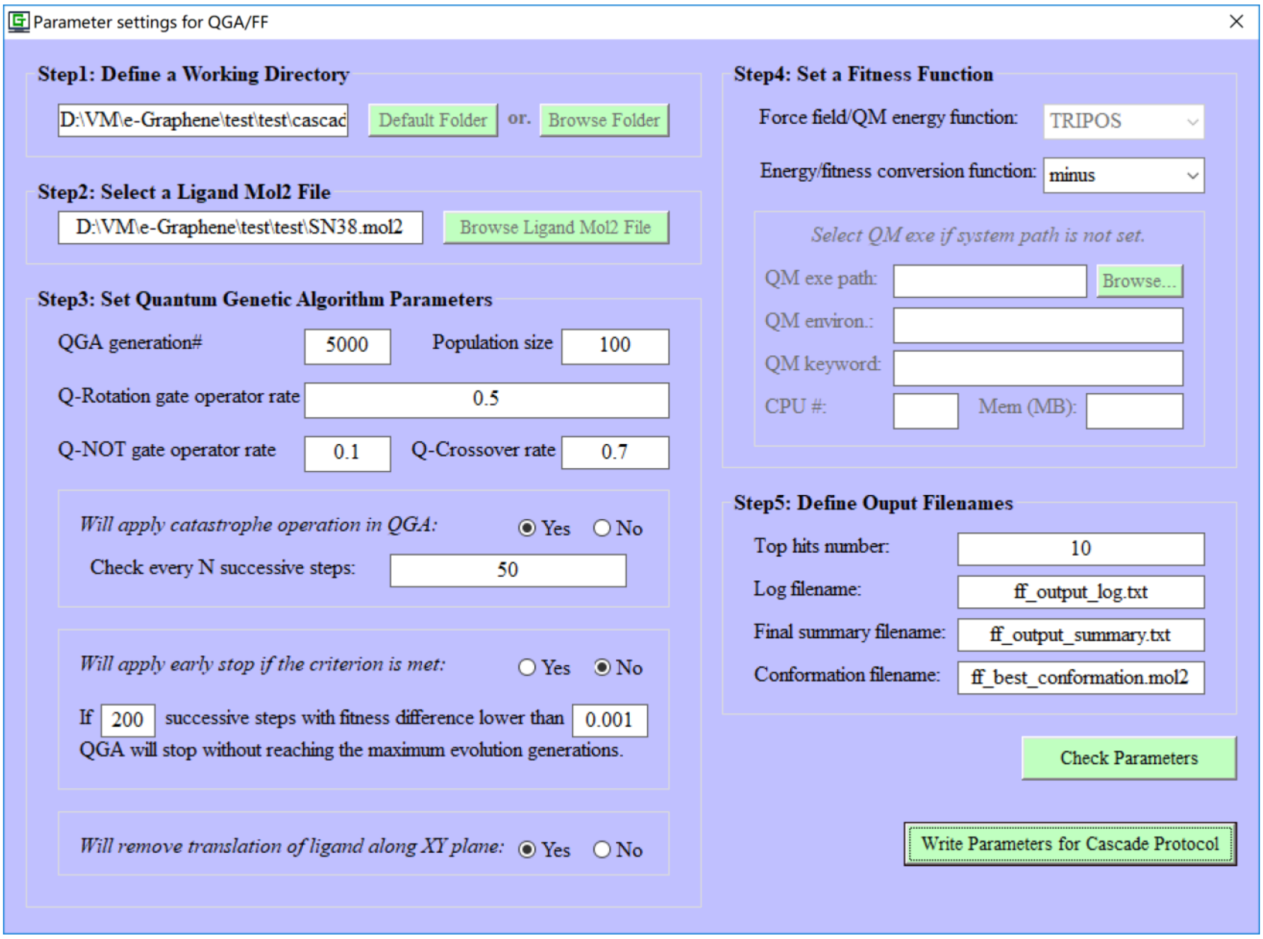

Figure S2. Parameter settings for QGA/FF in the two-layer cascade protocol.

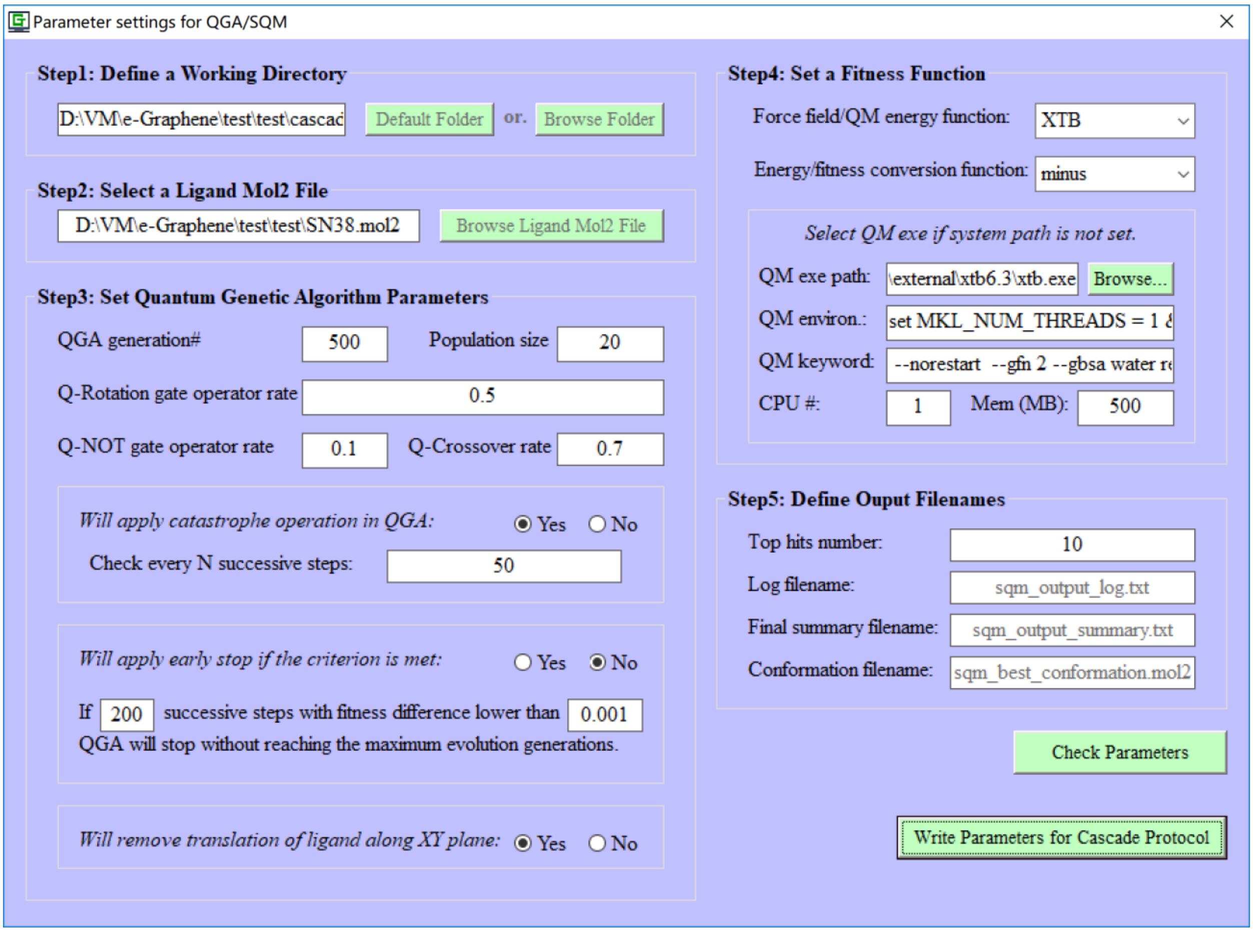

Figure S3. Parameter settings for QGA/SQM in the two-layer cascade protocol.

Table S1. The update scheme of quantum rotation angle

n_i	$best_i$	$f(n)<f(best)$	$\Delta\theta_i$	$\alpha_i\beta_i>0$	$\alpha_i\beta_i<0$	$\alpha_i=0$	$\beta_i=0$
0	0	False	0	0	0	0	0
0	0	True	0	0	0	0	0
0	1	False	0.03π	+1	−1	0	±1
0	1	True	0.01π	−1	+1	±1	0
1	0	False	0.03π	−1	+1	±1	0
1	0	True	0.01π	+1	−1	0	±1
1	1	False	0	0	0	0	0
1	1	True	0	0	0	0	0

Notes: (1) *best* and *n* refer to the best chromosome and n^{th} chromosome in the whole population; (2) $best_i$ and n_i refer to the collapsed ground state (|0> or |1>) after measurement of the ith qubit in the best chromosome and n^{th} chromosome; (3) *f(n)* and *f(best)* are the fitness score for the best and n^{th} chromosomes respectively; (4) α_i and β_i refer to the probability amplitudes of the ith qubit in the chromosome; (4) $\Delta\theta_i$ is the variation of quantum rotation angle for the ith qubit in the chromosome.

Table S2. GDDS (graphene/GOs-SN38) prediction results after three runs of cascade protocol.

Mode	Model type	N_e	N_h	N_c	Binding energy for repeat 1	Binding energy for repeat 2	Binding energy for repeat 3	Average binding energy over three repeats
M001	graphene	0	0	0	-25.6385	-25.4064	-26.3904	-25.8118
M002	GO-1	1	1	1	-26.1971	-25.5411	-26.8993	-26.2125
M003	GO-1	1	1	1	-28.7635	-26.6427	-23.6562	-26.3541
M004	GO-1	1	1	1	-26.4577	-26.1523	-26.2719	-26.2940
M005	GO-1	1	1	1	-27.4865	-27.6723	-27.7790	-27.6459
M006	GO-1	1	1	1	-23.8420	-23.7255	-21.7282	-23.0986
M007	GO-1	1	1	1	-24.1559	-23.5582	-25.8729	-24.5290
M008	GO-1	1	1	1	-24.9834	-26.0930	-28.1963	-26.4242
M009	GO-1	1	1	1	-24.7224	-25.6496	-24.9523	-25.1081
M010	GO-1	1	1	1	-26.6718	-27.5633	-26.6450	-26.9601
M011	GO-1	1	1	1	-25.6391	-23.0569	-25.9614	-24.8858
M012	GO-1	1	1	1	-26.7573	-24.3444	-26.7485	-25.9501
M013	GO-1	1	1	1	-28.7096	-27.6435	-28.2588	-28.2040
M014	GO-1	1	1	1	-22.8619	-23.0481	-28.7977	-24.9026
M015	GO-1	1	1	1	-26.6067	-26.5347	-25.7772	-26.3062
M016	GO-1	1	1	1	-25.2820	-27.4653	-27.8848	-26.8774
M017	GO-1	1	1	1	-22.3913	-21.5477	-19.9207	-21.2866
M018	GO-1	1	1	1	-25.5005	-25.3733	-23.2009	-24.6916
M019	GO-1	1	1	1	-27.4771	-27.4957	-26.0815	-27.0181
M020	GO-1	1	1	1	-23.3872	-28.7554	-24.5514	-25.5646
M021	GO-1	1	1	1	-26.9070	-26.6283	-27.0594	-26.8649
M022	GO-2	2	2	2	-21.4185	-22.1254	-21.6975	-21.7471
M023	GO-2	2	2	2	-21.1139	-20.0987	-25.5742	-22.2623
M024	GO-2	2	2	2	-28.7374	-26.1651	-28.4845	-27.7957
M025	GO-2	2	2	2	-24.6334	-25.3562	-25.2510	-25.0802
M026	GO-2	2	2	2	-25.4020	-24.2319	-25.6209	-25.0849
M027	GO-2	2	2	2	-27.1203	-27.4754	-24.6393	-26.4116
M028	GO-2	2	2	2	-25.8723	-26.5411	-26.7536	-26.3890
M029	GO-2	2	2	2	-27.9815	-27.5844	-28.6191	-28.0617
M030	GO-2	2	2	2	-25.8967	-26.0626	-25.9022	-25.9538
M031	GO-2	2	2	2	-25.4991	-26.0346	-27.6848	-26.4062
M032	GO-2	2	2	2	-26.7480	-25.8772	-27.9773	-26.8675
M033	GO-2	2	2	2	-29.3171	-25.7776	-27.9548	-27.6832
M034	GO-2	2	2	2	-27.2225	-25.5313	-26.9204	-26.5581
M035	GO-2	2	2	2	-22.2031	-22.7567	-21.4831	-22.1476
M036	GO-2	2	2	2	-24.6256	-15.1439	-24.4898	-21.4198
M037	GO-2	2	2	2	-23.7693	-27.9117	-26.0672	-25.9161
M038	GO-2	2	2	2	-28.4553	-28.8178	-24.9325	-27.4019
M039	GO-2	2	2	2	-18.6883	-19.9409	-20.5178	-19.7157
M040	GO-2	2	2	2	-21.6713	-22.8466	-26.7987	-23.7722
M041	GO-2	2	2	2	-21.4150	-21.8530	-21.4185	-21.5622

M042	GO-3	3	3	3	-24.6963	-27.1338	-21.9948	-24.6083
M043	GO-3	3	3	3	-25.3750	-25.9214	-22.1415	-24.4793
M044	GO-3	3	3	3	-24.5547	-20.6358	-21.2871	-22.1592
M045	GO-3	3	3	3	-24.4995	-25.0466	-25.2372	-24.9278
M046	GO-3	3	3	3	-21.3603	-17.5635	-21.5452	-20.1563
M047	GO-3	3	3	3	-23.7338	-17.0983	-23.8673	-21.5664
M048	GO-3	3	3	3	-26.1441	-26.4890	-25.3769	-26.0033
M049	GO-3	3	3	3	-25.4747	-24.4726	-25.5763	-25.1745
M050	GO-3	3	3	3	-22.9999	-22.4482	-16.9119	-20.7867
M051	GO-3	3	3	3	-22.7361	-22.9218	-21.9861	-22.5480
M052	GO-3	3	3	3	-25.0992	-23.6829	-27.3166	-25.3662
M053	GO-3	3	3	3	-22.5906	-23.9896	-24.2799	-23.6200
M054	GO-3	3	3	3	-22.3889	-18.4955	-27.4262	-22.7702
M055	GO-3	3	3	3	-28.5202	-27.6224	-28.9465	-28.3630
M056	GO-3	3	3	3	-15.8007	-18.4291	-15.0913	-16.4404
M057	GO-3	3	3	3	-27.6947	-22.6387	-27.3033	-25.8789
M058	GO-3	3	3	3	-23.4998	-24.0848	-24.2666	-23.9504
M059	GO-3	3	3	3	-24.3441	-25.1212	-24.7888	-24.7514
M060	GO-3	3	3	3	-25.6392	-21.1625	-19.1251	-21.9756
M061	GO-3	3	3	3	-21.7726	-21.9998	-21.7438	-21.8387
M062	GO-4	4	4	4	-26.0763	-23.7978	-24.7587	-24.8776
M063	GO-4	4	4	4	-15.2881	-15.8854	-14.3562	-15.1766
M064	GO-4	4	4	4	-23.0809	-28.0997	-17.6126	-22.9311
M065	GO-4	4	4	4	-29.6982	-26.3668	-28.1731	-28.0794
M066	GO-4	4	4	4	-19.3796	-23.2222	-23.0079	-21.8699
M067	GO-4	4	4	4	-18.8141	-19.1936	-21.3533	-19.7870
M068	GO-4	4	4	4	-19.3712	-19.1406	-20.2241	-19.5786
M069	GO-4	4	4	4	-14.5083	-18.8300	-17.5951	-16.9778
M070	GO-4	4	4	4	-21.4509	-22.5838	-26.1379	-23.3909
M071	GO-4	4	4	4	-26.8721	-24.4955	-25.2565	-25.5414
M072	GO-4	4	4	4	-25.2715	-27.8149	-27.6965	-26.9276
M073	GO-4	4	4	4	-19.9009	-14.4564	-14.0141	-16.1238
M074	GO-4	4	4	4	-31.7920	-18.5326	-16.4403	-22.2550
M075	GO-4	4	4	4	-17.1811	-17.1349	-16.3046	-16.8735
M076	GO-4	4	4	4	-27.7511	-20.6038	-17.0905	-21.8151
M077	GO-4	4	4	4	-17.2248	-19.2634	-19.8037	-18.7639
M078	GO-4	4	4	4	-25.3845	-25.2387	-24.9849	-25.2027
M079	GO-4	4	4	4	-28.9551	-29.2431	-27.8880	-28.6954
M080	GO-4	4	4	4	-27.2964	-29.0332	-22.1568	-26.1621
M081	GO-4	4	4	4	-19.8038	-15.9950	-19.7941	-18.5310
M082	GO-5	5	5	5	-17.4887	-20.8910	-18.2345	-18.8714
M083	GO-5	5	5	5	-17.9410	-22.6450	-22.3368	-20.9743
M084	GO-5	5	5	5	-17.1349	-16.1277	-18.9360	-17.3995
M085	GO-5	5	5	5	-25.8445	-12.5535	-13.0713	-17.1564
M086	GO-5	5	5	5	-9.4649	-15.2452	-17.0707	-13.9269
M087	GO-5	5	5	5	-22.4236	-20.2077	-16.5766	-19.7360
M088	GO-5	5	5	5	-17.4711	-27.5495	-16.2042	-20.4083
M089	GO-5	5	5	5	-20.7044	-18.8648	-22.9814	-20.8502
M090	GO-5	5	5	5	-22.1457	-27.7093	-21.7021	-23.8524

M091	GO-5	5	5	5	-13.5838	-13.0390	-20.3611	-15.6613
M092	GO-5	5	5	5	-13.4658	-24.2851	-19.2208	-18.9906
M093	GO-5	5	5	5	-20.3051	-20.0050	-17.5068	-19.2723
M094	GO-5	5	5	5	-23.8201	-21.8417	-21.1040	-22.2553
M095	GO-5	5	5	5	-26.0499	-19.0153	-17.6567	-20.9073
M096	GO-5	5	5	5	-16.9065	-19.9376	-18.2565	-18.3669
M097	GO-5	5	5	5	-25.8755	-26.5125	-26.8271	-26.4050
M098	GO-5	5	5	5	-20.2694	-23.4175	-19.1355	-20.9408
M099	GO-5	5	5	5	-19.1211	-22.0218	-18.9799	-20.0409
M100	GO-5	5	5	5	-22.8683	-25.9316	-25.5950	-24.7983
M101	GO-5	5	5	5	-27.2076	-27.4873	-23.4700	-26.0550

(1) N_e, N_h, N_c are the number of epoxy, hydroxyl and carboxyl groups respectively; (2) GO-1, GO-2, GO-3, GO-4 and GO-5 stand for five different modification rates on the pristine graphene model.

Table S3. GDDS screen results after three runs of cascade protocol.

DrugBank ID	Binding energy between graphene and drug molecule for the first repeat of screen	Binding energy between graphene and drug molecule for the second repeat of screen	Binding energy between graphene and drug molecule for the third repeat of screen	Average binding energy over three repeats of screen
DB04711	-40.2132	-34.9615	-33.9092	-36.3613
DB14201	-35.0988	-29.7986	-34.3159	-33.0711
DB06595	-31.9577	-31.9824	-31.7168	-31.8856
DB11433	-32.8532	-28.8593	-32.9876	-31.5667
DB00799	-30.4970	-31.0882	-30.9945	-30.8599
DB04868	-32.4640	-27.9260	-32.0384	-30.8095
DB11652	-29.9327	-32.7242	-26.4233	-29.6934
DB11399	-30.1040	-26.6340	-30.0629	-28.9337
DB13766	-28.3067	-28.8713	-29.0842	-28.7541
DB00735	-27.7617	-29.5548	-28.7084	-28.6750
DB06249	-23.5976	-30.6208	-29.2726	-27.8303
DB01022	-27.3787	-29.6582	-25.8082	-27.6150
DB00210	-25.5552	-28.7235	-26.6373	-26.9720
DB13953	-26.0870	-25.9500	-26.9871	-26.3413
DB11585	-26.8295	-23.3080	-28.2686	-26.1354
DB15444	-26.2079	-24.7926	-27.2462	-26.0823
DB09030	-25.7021	-25.7153	-25.9080	-25.7751
DB09143	-26.9172	-25.0394	-25.0492	-25.6686
DB00459	-28.0543	-21.2565	-26.5119	-25.2743
DB13946	-24.8268	-26.7390	-23.6110	-25.0589
DB01012	-26.5225	-21.7721	-26.8805	-25.0584
DB09340	-23.1244	-26.1561	-25.6165	-24.9657
DB00872	-24.5249	-23.7323	-25.9080	-24.7217
DB08804	-25.0707	-22.6070	-26.1775	-24.6184
DB04842	-23.1285	-25.0954	-25.5859	-24.6033
DB08912	-23.7909	-22.3853	-27.4446	-24.5403
DB13277	-24.1787	-24.2233	-24.4806	-24.2942
DB01091	-23.7647	-24.1599	-24.9278	-24.2842
DB00857	-23.2015	-24.8463	-24.5689	-24.2056
DB09496	-24.8061	-21.5030	-26.2518	-24.1870
DB08822	-21.5905	-21.9875	-28.5273	-24.0351
DB11986	-21.2933	-24.2578	-26.2285	-23.9265
DB00796	-22.6538	-25.2629	-23.7724	-23.8964
DB13943	-23.8093	-23.9045	-23.4552	-23.7230
DB00679	-22.6702	-24.4057	-23.7988	-23.6249
DB04841	-24.2735	-22.6734	-23.9061	-23.6177
DB11742	-25.8951	-23.1136	-21.6647	-23.5578
DB00549	-23.5643	-23.2670	-23.6482	-23.4932
DB11226	-20.8153	-26.1171	-23.0670	-23.3331
DB14639	-23.8453	-23.9357	-22.1792	-23.3200

DB12808	-25.1119	-20.6372	-24.1547	-23.3013
DB00568	-22.2721	-23.3488	-23.9817	-23.2009
DB08827	-21.3644	-22.6417	-25.2550	-23.0870
DB00932	-21.2655	-19.6491	-28.3454	-23.0867
DB01134	-22.9414	-22.9947	-22.5213	-22.8191
DB01029	-22.7864	-23.0998	-22.1653	-22.6838
DB04794	-21.0292	-23.3047	-23.5960	-22.6433
DB08864	-20.8003	-24.8260	-22.1446	-22.5903
DB13944	-21.9636	-22.7810	-22.6140	-22.4529
DB04825	-21.8858	-25.0854	-20.0423	-22.3378
DB14002	-20.8275	-22.7465	-22.0118	-21.8619
DB00163	-17.5816	-27.4004	-20.3119	-21.7646
DB09238	-19.3177	-19.4576	-26.2737	-21.6830
DB00637	-22.2200	-22.5196	-19.9617	-21.5671
DB08820	-23.4985	-19.8394	-20.9839	-21.4406
DB00984	-21.7255	-20.9775	-21.3211	-21.3414
DB00528	-20.5092	-19.4974	-23.9881	-21.3316
DB11570	-21.2856	-20.2764	-22.4131	-21.3250
DB00966	-21.8726	-21.3286	-20.2866	-21.1626
DB11501	-23.7002	-23.7021	-15.9478	-21.1167
DB11460	-21.3862	-21.7432	-20.1099	-21.0798
DB00661	-23.7554	-18.2822	-20.9555	-20.9977
DB05239	-20.7742	-21.4296	-20.7616	-20.9885
DB00197	-21.7671	-20.5038	-20.6551	-20.9754
DB08887	-17.1947	-21.4330	-23.9361	-20.8546
DB00982	-20.9832	-21.8439	-19.3393	-20.7221
DB00159	-19.7163	-20.0977	-21.8839	-20.5660
DB00523	-18.0716	-21.8628	-21.6637	-20.5327
DB13966	-17.7300	-22.1229	-21.0047	-20.2859
DB14655	-20.0814	-20.0064	-20.2358	-20.1079
DB11062	-19.3250	-20.6314	-20.3047	-20.0871
DB13956	-19.9228	-19.6156	-20.3184	-19.9523
DB13954	-20.5061	-19.6040	-18.9370	-19.6824
DB09280	-18.6398	-20.1923	-19.2025	-19.3449
DB11219	-18.7627	-19.1989	-19.1449	-19.0355
DB00673	-18.5400	-19.2619	-18.8742	-18.8920
DB01076	-16.1916	-21.0371	-19.2873	-18.8386
DB00675	-19.3357	-19.1108	-17.6310	-18.6925
DB00342	-18.2294	-19.5320	-18.1887	-18.6500
DB11517	-19.0590	-19.1442	-17.6212	-18.6081
DB00177	-19.1826	-18.4688	-17.7321	-18.4612
DB01070	-19.9459	-14.9281	-20.4597	-18.4446
DB14476	-17.8363	-19.6077	-17.8086	-18.4175
DB00392	-18.7246	-18.2405	-18.2433	-18.4028
DB00307	-18.5136	-18.2719	-18.4207	-18.4021
DB06410	-18.3926	-17.6918	-18.4619	-18.1821
DB06401	-15.4661	-21.0665	-17.1715	-17.9014
DB00132	-16.6457	-18.8821	-18.1169	-17.8816
DB11201	-17.2098	-17.5198	-18.7520	-17.8272

DB11269	-19.1589	-15.8967	-18.0400	-17.6985
DB06202	-19.0082	-17.0398	-16.6949	-17.5809
DB00146	-17.5487	-16.6459	-18.1586	-17.4511
DB00470	-18.2230	-18.2659	-15.2949	-17.2613
DB04224	-18.0794	-17.6564	-15.4815	-17.0724
DB01410	-14.8124	-18.4877	-17.8449	-17.0483
DB06702	-15.6819	-19.7293	-15.6819	-17.0310
DB14104	-16.3017	-18.7375	-16.0287	-17.0227
DB11495	-15.7885	-16.2437	-18.9056	-16.9793
DB13854	-16.6987	-15.8440	-17.0911	-16.5446
DB01436	-17.3993	-14.2443	-17.3330	-16.3255
DB09056	-16.4197	-16.3915	-16.1127	-16.3079
DB09016	-16.2773	-15.8767	-15.7561	-15.9701
DB09061	-15.0292	-16.5216	-16.1922	-15.9144
DB11328	-16.3637	-16.0047	-14.3136	-15.5607
DB11431	-13.7655	-15.9586	-16.3948	-15.3730
DB06789	-14.6359	-15.6328	-15.5790	-15.2826
DB12728	-16.4795	-14.4004	-14.8273	-15.2357
DB11260	-15.3405	-15.1597	-15.0744	-15.1916
DB08958	-15.1250	-14.9019	-15.5468	-15.1913
DB14677	-15.2290	-15.3492	-14.7291	-15.1024
DB09048	-14.8703	-15.1389	-15.2587	-15.0893
DB11936	-13.5041	-12.5292	-17.0923	-14.3752
DB11427	-14.2808	-14.7112	-13.1550	-14.0490
DB11423	-7.8443	-11.3541	-21.9529	-13.7171
DB00255	-12.4285	-13.3472	-14.7475	-13.5078
DB09291	-13.1790	-13.2339	-13.7230	-13.3787
DB00486	-13.3561	-12.0129	-13.9776	-13.1155
DB00333	-10.5853	-13.9265	-12.9168	-12.4762
DB14555	-12.2615	-12.3759	-12.3759	-12.3378
DB00834	-12.3200	-12.3844	-12.2709	-12.3251
DB11262	-12.6626	-10.7653	-11.7509	-11.7263

This is the end of supporting information.
